# Effect of low back pain on clinical-functional factors and its associated potential risk of chronicity in adolescent dancers of classical ballet: cross-sectional study

**DOI:** 10.1186/s13102-022-00474-6

**Published:** 2022-05-02

**Authors:** Brenda Luciano de Souza, Patricia Colombo de Souza, Ana Paula Ribeiro

**Affiliations:** 1 Biomechanics and Musculoskeletal Rehabilitation Laboratory, Health Science Post-Graduate Department, School of Medicine, University Santo Amaro, R. Professor Enéas de Siqueira Neto, 340, Campus I, São Paulo, SP 04829-900 Brazil; 2 Physical Therapy and Sport Science Department, School of Medicine, University of São Paulo, São Paulo, Brazil

**Keywords:** Young dancers, Pain, Lumbar, Spine flexion, Foot, Posture, Chronic back pain

## Abstract

**Background:**

Low back pain (LBP) is a common symptom in classical ballet dancers, which can limit their daily activities and dance training routines. The purpose of the study was to verify the association and comparison of clinical-functional outcomes (spine flexibility and foot posture) between different levels of intensity low back pain in adolescents of classical ballet and the potential risk of chronicity using the STarT back tool.

**Methods:**

Cross-sectional study. Participants: 78 adolescent girls who practice classical ballet were evaluated and divided into groups according to level of low back pain: mild (n = 21), moderate (n = 17), and high (n = 20), and a control group (n = 20). Main outcome measures: Pain, flexibility of the spine (thoracic and lumbosacral), risk of chronicity for low back pain, and foot posture were assessed using the visual analogue scale, clinical tests, STarT back screening tool (SBST) questionnaire, and foot posture index (FPI), respectively.

**Results:**

Dancers with high-intensity low back pain showed a potential risk of chronicity by the SBST. The spine pain intensity was not different considering thoracic and lumbosacral flexibility in the sagittal plane, but was different with greater supine FPI when compared to control dancers. Mild low back pain was associated with greater supine FPI. The SBST score was associated with higher exposure time–frequency and time of dancing.

**Conclusion:**

Adolescents of classical ballet with high-intensity low back pain showed a potential risk of chronicity by the SBST. The level of intensity low back pain did not influence the clinical-functional aspects of spine flexibility in the sagittal plane, but the level of intensity moderate pain promoted changes in foot posture (more supinated). The potential risk of chronicity using the SBST was also associated with higher exposure time–frequency and time of dancing, in adolescents of classical ballet.

## Background

Low back pain is a common musculoskeletal disorder in pre-professional and professional dancers, with 78% of dancers reporting at least one episode of low back pain over a period of 1 year [1]. Low back pain is linked to a dancer’s volume of training; occurring per 1000 h of practice (back pain/1000 h practice: exposure time) [2], which limits their daily activities and dance training routines [1]. Moreover, 24% of dancers have experienced chronic low back pain, with the highest prevalence rate observed in the previous 3 months, 17% of which interrupted dance activities [1].

The prevalence of low back pain is attributed to the complex movements in ballet practice, which involve rapid changes in direction, jumps, and turns, requiring strength [3], flexibility [4], great amplitudes of articular movements [5, 6], and extreme postural control through the foot support base [7]. The repetitive joint movement of the spine, in particular, is associated with strength overload and possible postural adjustments [8, 9]. However, the etiology of low back pain remains multifactorial [10, 11], with 90% of cases being nonspecific (no clearly defined cause) [1, 11, 12].

Previous studies have been shown that the lumbar musculature is always highly recruited during spine hyperextension and hip extension in ballet [6]. Other studies reveal that in dancers, abdominal musculature that cannot support the lumbar region can compromise the flexibility between the muscular chains: the anterior and posterior spine [13]. This results in a marked lumbar curvature (lumbar hyperlordosis), thereby contributing to the vicious cycle of asymmetry between the lumbar muscles and the abdominal muscles [13, 14], which in turn result in possible changes in the support planting of the feet [15]. Healthy dancers, that is, those without symptoms of low back pain, present significant biomechanical alterations in foot support, thus promoting marked increases in the plantar overload on the forefoot [16] and the lumbosacral spine [17]. Another noteworthy aspect is the lack of plantar support over the midfoot region; due to dance practice, which results in changes in body balance that lead to great mid-lateral oscillations [18, 19]. Some studies focusing on postural adjustments reveal that dancers present increased lumbar lordosis, pelvic tilt [9], varus knees [20], and foot pronation [21].

During adolescence, physical changes in dancers can increase vulnerability to spinal symptoms and injuries or future musculoskeletal injury, due to high movement repetitions, at varying velocities, and an intense training program [10, 22, 23]. Thus, the challenge is to understand re-injury and the risk of developing chronic low back pain in dancers, in order to be able to direct the prognosis [3, 24]. Together with the difficulty of monitoring chronic low back pain, understanding the potential risk factors for prognosis becomes even more challenging during constant dance practice [11, 25]. One of the risks of chronicity for low back pain in adolescents of classical ballet is identification of the biopsychosocial risk factors involved in its development [11, 26]. However, the dance and health professionals involved in preventive and conservative treatment of low back pain in adolescents do not sufficiently understand the clinical-functional aspects and biopsychosocial risk factors for its evolution, especially in dancers of classical ballet [3].

The STarT Back Screening Tool (SBST) questionnaire is an important tool for assessing the risk of chronicity for low back pain through measures of modifiable biopsychosocial factors, with precision and validity already proven in patients with acute/chronic low back pain with or without physical therapy treatment [25, 26]. In sport, the SBST is used to monitor changes in a range of modifiable prognostic factors in patients with low back pain undergoing different levels of physical therapy treatment [27]. This tool offers a great clinical advantage as it is quick, simple, and easy to understand and apply by health professionals [25]. The SBST questionnaire is used by health professionals to identify the risk of chronicity for low back pain through modifiable biopsychosocial factors, thus better targeting and supporting the preventive and conservative treatment of compromised dancers; consequently, aiding the prevention of future functional losses and musculoskeletal injuries, which can lead to withdrawals and interruptions of dance practice [25, 28]. Despite these benefits, there are still no studies focusing on the application of this questionnaire in adolescents practicing classical ballet. Thus, the objective of this study was to verify the association and comparison of clinical-functional outcomes (spine flexibility and foot posture) between different levels of intensity low back pain in adolescents of classical ballet and the potential risk of chronicity using the STarT back tool.

## Methods

### Study type and sample selection

Analytical survey: a cross-sectional study involving 78 adolescent girls practicing intermediate and advanced classical ballet as members of the dance company Ballet Paraisópolis, which is located in the western region of São Paulo. The adolescents were divided into three groups according to their level of intensity persistent symptoms of low back pain in the previous 14 consecutive days, during a 3-month period of persistent pain symptoms (group 1: adolescents with mild low back pain [0–3 pain intensity, n = 21]; group 2: adolescents with moderate low back pain [4–6 pain intensity, n = 17]; group 3: adolescents with high low back pain [7–10 pain intensity, n = 20]) following the guidelines of the Spine Society [37]. The control group consisted of 20 adolescent dancers of classical ballet who did not have any symptom of low back pain.

The study procedure was reviewed and approved by the Departmental Research Committee of the University Santo Amaro-UNISA (registration number: 3.756.991), in accordance with the Helsinki Declaration and relevant guidelines and regulations. Prior to participation, all volunteers were provided with information about the study and given the opportunity to ask questions before providing written informed consent/assent, as well as parental consent when required.

The eligibility criteria were as follows: adolescents of classical ballet with and without low back pain, adolescents who indicated that they had pain (current) in the region of the lower back with constant symptoms in the previous 14 consecutive days, during a 3-month period of pain symptoms, minimum age of 12 years [29], regular enrollment in Ballet Paraisópolis, with a twice-a-week training frequency [30], and 1-year practice time [32, 38]. The exclusion criteria were as follows: pain with irradiation to the lower limbs, medication use for pain (current), neurological abnormalities [24], congenital diseases [28, 30], spinal disorders [29], and the presence of tumors (cancer) or infection [30]. In addition, fractures in the previous 6 months; vestibulocochlear diseases; uncontrolled cardiac and/or respiratory arrhythmias; convulsive and neurological syndromes; diabetic neuropathy, osteoarthritis and rheumatoid arthritis, and any functional limitation that requires assistance in moving around were regarded as exclusion criteria to avoid bias in the interpretation of the assessments [31].

### Initial evaluation

The initial questionnaire was applied to collect information on anthropometric characteristics (age, height, body mass, and body mass index) and Classical Ballet sports practice (time and frequency of training, as well as years of practice and musculoskeletal injuries) [12, 30].

### Evaluation of low back pain and prognosis by STarT back screening tool (SBST)

The symptom of low back pain was assessed using the visual analogue scale (VAS); the scale is from 0 to 100 mm, with 0 indicating absence of pain and 100 unbearable pain [32].

For stratification of low back pain prognosis, the SBST questionnaire was used, which has shown reliability and validity [25]. This tool classifies a patient regarding the risk of chronicity for low back pain, with modifiable biopsychosocial risk factors. The questionnaire contains nine items related to low back pain; items 1–4 are related to referred pain, dysfunction, and comorbidities (such as shoulder and neck pain), and items 5–9 are related to psychosocial changes (referring to discomfort, catastrophizing, fear, anxiety, and depression). A score on this subscale of ≤ 3 points indicates medium risk, and > 3 points indicates high risk [33, 34]. Thus, the classifications adopted were; high risk when demonstrating a high level of psychosocial factors with or without the presence of physical factors, medium risk when demonstrating a low level of physical and psychosocial factors, and low risk when demonstrating a minimum level of physical and psychosocial factors [33].

### Spine assessment: thoracic and lumbosacral flexibility

The Schober clinical test was performed to assess the flexibility of the thoracic and lumbosacral spine on sagittal plane during anterior trunk flexion [46]; with previously demonstrated validity and reliability [35, 36]. To perform this test, each individual was barefoot; and the region of the lumbosacral spine was free of clothing to enable the examining physiotherapist to delimit the anatomical points. With a ballpoint pen, the physiotherapist marked the lower margin of the posterior and superior iliac spines by drawing a horizontal line on the midline between these two anatomical points. Then, the physiotherapist then positioned the tip of a tape measure firmly against the epithelial tissue of the region with the marked outline; and a second, vertical mark was added 15 cm above the initial mark. The participant was asked to flex the anterior torso until the onset of pain, and a new measurement was marked between the demarcations (lower and upper); the adolescent then returned to the neutral position. The difference between the initial distance (between the two demarcations on the skin in the neutral position) and the new measurement in the flexed position of the trunk was used to indicated the flexibility (mobility) of the lumbosacral spine in centimeters, with millimeter-level precision. This measure, which initially is 15 cm initially in the orthostatic position, should be increased by 6 cm during trunk flexion. After measurement, the marks were removed with alcohol gel [37].

The Stibor Index measures the flexibility between the segments of the thoracic and lumbosacral spines. With a ballpoint pen, the physiotherapist drew a line along the spinous process of the seventh cervical vertebra (C7) and the fifth lumbar vertebra (L5–S1), which was previously demarcated. With the tape measure, the distance between the two anatomical points was measured and then demarcated. The participant was then asked to perform anterior trunk flexion, and the examiner again measured the distance between the two points. The Stibor Index represents the difference between the two markings (in the orthostatic and inclined positions). For individuals with normal flexibility, this point should move to indicate an increase in distance of approximately 10 cm [38].

### Foot posture assessment: foot posture index (FPI)

Foot posture assessment was performed using the foot posture index (FPI), a clinical diagnostic tool designed to quantify the degree to which the foot can be considered supine, pronated, or normal. The participant was positioned in the orthostatic position with bipedal support; a 7.5 cm rectangle was placed between their feet for greater standardization of the plantar surface support base. In addition, the individual was instructed to position their upper limbs along the trunk with their gaze directed forward. All participants were instructed to perform this position, since any movement or inclination of the body would significantly alter the results. Each participant was graded as 0 (neutral), + 1 or + 2 (pronated), or − 1 or − 2 (supinated) [39, 40].

### Statistical analysis

All statistical analyses were performed using SPSS version 24 (IBM, Chicago, IL, USA). The normality of the data was verified using the Shapiro–Wilk test. To compare the measurements of the dependent variables between the groups with low back pain, one-way analysis of variance (ANOVA) was used for the independent measures, followed by Tukey’s post-hoc. Multiple linear regression analysis was used to verify the relationship between the SBTS score of each group with low back pain and the symptom of pain, time of dance practice, and frequency of weekly training were adjusted in the models. For all analyses, significant differences were considered when *p* < 0.05.

## Results

The anthropometric variables of age, height, body mass, and body mass index did not show statistically significant differences between the groups: group 1 (mild low back pain), group 2 (moderate low back pain), and group 3 (high low back pain). Only the frequency of weekly dance training and the percentage of injuries significantly differed between the groups with low back pain in relation to the control group; the group with high low back pain (CI 15.3–23.6, *p* = 0.010) presented higher values compared with those of the control group (CI 8.8–20.6, *p* = 0.010) and differences low back pain high and mild with high (CI 9.8–16.7, *p* = 0.010) and with moderate low back pain (CI 10.6–20.2, *p* = 0.010) (Table 1). This demonstrates that physical effort can affect the level of low back pain.

**Table 1:** Mean, standard deviation, and comparisons between different groups of low back pain (mild, moderate, and high) in relation to control for anthropometric characteristics and dance activities of adolescent dancers of classical ballet

Anthropometric variables	Mild low back pain (1)		Moderate low back pain (2)		High low back pain (3)		Control group (4)		*p*
	Mean ± SD	95% CI	Mean ± SD	95% CI	Mean ± SD	95% CI	Mean ± SD	95% CI	
Age (years)	15.0 ± 2.2	11.8–15.8	16.0 ± 1.3	14.7–16.4	15.5 ± 2.1	14.8–16.5	14.6 ± 2.1	13.7–15.8	0.116
Height (m)	1.6 ± 0.7	1.53–1.7	1.6 ± 0.6	1.55–1.65	1.6 ± 0.9	1.58–1.64	1.6 ± 0.7	1.57–1.62	0.980
Body mass (kg)	52.6 ± 8.5	41.5–60.0	54.6 ± 7.5	48.9–57.0	50.7 ± 6.8	48.3–55.8	50.5 ± 8.5	48.8–54.7	0.266
Body mass index (kg/m^2^)	20.0 ± 2.6	17.8–21.4	20.8 ± 2.6	19.6–21.5	19.5 ± 1.9	18.8–20.9	19.3 ± 2.7	19.1–21.2	0.310
Practice time (years)	9.2 ± 3.1	6.4–12.2	7.6 ± 3.5	8.0–11.6	7.7 ± 4.2	6.5–12.8	8.6 ± 3.8	6.6–11.4	0.503
Training frequency (h/week)	14.7 ± 11.8	9.8–16.7	14.2 ± 9.9	10.6–20.2	19.8 ± 10.0	15.3–23.6	13.9 ± 9.5	8.8–20.6	0.010^&^*^#^
Musculoskeletal injuries (%)	50%	–	75%	–	88%	–	40%	–	0.001^&^*^#^

*One-way ANOVA, followed by Tukey’s post-hoc, considering statistical differences *p* < 0.05

^&^Differences low back pain high and mild

*Differences low back pain high and moderate

^#^Differences low back pain high and control

Table 2 shows that the risk of chronicity for low back pain was greater in the group of adolescents of classical ballet with high low back pain (CI 0.8–2.8, *p* = 0.008) when compared to adolescent with mild and moderate low back pain and the control group (*p* > 0.008). Another important finding was that the dancers with different intensities of low back pain (low back pain), in relation to the control group did not differ between the Schober and Stibor tests, showing that the flexibility of the thoracic and lumbosacral spine is not influenced by pain in adolescents of classical ballet. In addition, the FPI on both the right and left sides, was more supine in the group with mild back pain (CI − 0.12 to 3.4, *p* = 0.010 right foot and CI − 0.11 to 3.2, *p* = 0.010 left foot) compared to the dancers with moderate and high back pain and the control group.

**Table 2:** Mean, standard deviation, and comparisons between different groups of low back pain (mild, moderate, and high) in relation to control for Start Back Screening Tool (SBST), clinical tests by Schober and Stibor, and foot posture index (FPI) of adolescent dancers of classical ballet

Physical exam	Mild low back pain (1)		Moderate low back pain (2)		High low back pain (3)		Control group (4)		*p*
	Mean ± SD	95% CI	Mean ± SD	95% CI	Mean ± SD	95% CI	Mean ± SD	95% CI	
SBST (score)	1.9 ± 1.1	0.8–2.8	2.8 ± 1.3	1.7–2.6	3.5 ± 1.4	2.9–4.4	–	–	0.008^&^*^#^
Schober test (cm)	12.5 ± 2.7	8.9–16.6	12.0 ± 2.6	10.7–12.9	13.4 ± 2.4	12.5–14.5	11.9 ± 2.9	10.4–12.3	0.335
Stibor test (cm)	15.9 ± 4.8	10.6–20.6	16.2 ± 3.2	14.1–17.6	16.7 ± 4.5	14.7–18.0	16.4 ± 4.4	13.8–17.3	0.946
Right FPI (score)	2.1 ± 4.7	− 0.7 to 6.5	1.0 ± 3.3	− 0.12 to 3.4	0.8 ± 3.0	− 1.2 to 1.8	0.6 ± 3.3	− 2.3 to 1.0	0.042^&^*^#^
Left FPI (score)	2.2 ± 3.6	− 0.5 to 6.7	0.5 ± 2.9	− 0.11 to 3.2	0.6 ± 3.4	− 1.6 to 1.4	0.4 ± 3.2	− 1.4 to 1.2	0.020^&^*^#^

*One-way ANOVA, followed by Tukey’s post-hoc, considering statistical differences *p* < 0.05

^&^Differences low back pain high and mild

*Differences low back pain high and moderate

^#^Differences low back pain high and control

In the multiple linear regression analysis, it can be observed that the SBST score was related to the pain symptom for all groups of low back pain (mild, moderate and high) showing that the greater the intensity of lower back pain, the worse the risk of chronicity of low back pain, with a greater and significant associative relationship in the groups of adolescents of classical ballet with moderate and high low back pain (Table 3).

**Table 3:** Simple linear regression showing the relationship between SBST score and level of low back pain in each group of adolescent classical ballet dancers with low back pain (mild, moderate, and high)

Pain level (groups)	SBST (score)	Pain (mm)	R	R^2^adjusted	T	*p*
Mild low back pain	1.9 ± 1.1	40.6 ± 0.60	0.26	0.02	15.9	0.001*
Moderate low back pain	2.8 ± 1.3	60.3 ± 0.50	0.46	0.16	21.0	< 0.001*
High low back pain	3.5 ± 1.4	80.5 ± 0.80	0.42	0.13	17.1	0.001*

*Multiple linear regression analysis model, considering statistical differences *p* < 0.05

Table 4 presents the multiple linear regression, showing that the higher the SBST score for poor low back pain, the greater the years of practice and intensity of dance training performed by adolescents. Thus, the SBST can be considered an effective tool for monitoring low back pain.

**Table 4:** Simple linear regression showing the relationship between SBST score of each group of adolescent classical ballet dancers with low back pain (mild, moderate, and high), time of dance practice, and frequency of weekly training

Pain level (groups)	SBTS (score)	Practice time (years)	Training frequency (h/week)	R	R^2^adjusted	T	*p*
Mild low back pain	1.9 ± 1.1	9.2 ± 3.1	14.7 ± 11.8	0.54	0.22	3.76	0.013*
Moderate low back pain	2.8 ± 1.3	7.6 ± 3.5	14.2 ± 9.9	0.71	0.43	3.11	0.008*
High low back pain	3.5 ± 1.4	7.7 ± 4.2	19.8 ± 10.0	0.33	0.02	2.37	0.029*

*Multiple Linear Regression analysis model, considering statistical differences *p* < 0.05

## Discussion

The present study aimed to verify the association and comparison of clinical-functional outcomes (spine flexibility and foot posture) between different levels of intensity low back pain in adolescents of classical ballet and the potential risk of chronicity by the STarT back tool. The main results demonstrated that the adolescents of classical ballet with high-intensity low back pain showed a potential risk of chronicity by the SBST. Another essential finding was that the spine pain intensity was not different for thoracic and lumbosacral flexibility (sagittal plane), but was higher with greater supine FPI when compared to control dancers. The SBST score was associated with greater exposure time–frequency and time of dancing.

The literature reveals that a clinical framework of low back pain negatively impacts the physical-functional aspects of dancers when the symptoms evolve to the chronic phase, which is related to higher rates of withdrawals from dance practice of dance, especially among professional and elite dancers [1, 41]. In addition, the SBST was significantly associated with low back pain in adolescents of classical ballet. [42]. In this study, the importance of the SBST questionnaire was verified for the early detection of risk of chronicity of low back pain in adolescents who practice classical ballet. This tool could improve the effectiveness of assessment in relation to the risk of transition from the symptomatic framework to the chronic phase, thereby preventing dancers from neglecting their practice.

The effectiveness of the SBST questionnaire for evaluating the risk of chronicity for low back pain in adolescent dancers could aid health professionals to develop more pragmatic preventive strategies according to the levels of low back pain, thus alleviating symptoms and the appearance of future musculoskeletal injuries, such as spondylolisthesis and herniated disc. According to Swain et al. [2], for adolescents of classical ballet, the more effective the prognosis for low back pain, the greater the chances of promoting efficient preventive strategies during dance.

Furthermore, the symptom of pain in dancers can cause emotional and behavioral changes [43] resulting in negative beliefs about disabilities in their dance performance [44]. In this context, the SBST's advantage was considering the risk of chronicity for low back pain and the psychosocial factors involved in the symptomatic clinical framework of the dancers, thus enabling the detection of, or identifying the need for a more specific intervention, considering the emotional aspects of the affected dancers. In addition to the emotional aspects, the physical-functional aspects are of great importance for dancers, with spine flexibility being a major functional requirement for the physical performance of dancers [45]. According to the literature, postural and muscular adaptations are performed by dancers to maintain body stability and spine flexibility; the most common adaptations are hyperlordosis and spine extension movement imposed during Ballet practice [6, 12, 13], which can exert excessive force on the lumbosacral spine, reducing the strength of the abdominal muscles [13] and reflecting on the reduction in flexibility of the spine (sagittal plane), as well as indirectly on the lower kinetic chain. In this rationale, some studies infer laterality asymmetry on the strength of trunk and abdominal muscles in adolescents of classical ballet with low back pain [13, 14], and that ballet dancers without low back pain, had more flexible hamstrings and hip flexors than controls [4]. Thus, the decreased hamstring flexibility, due to lumbar pain, could lead to increased lumbar overload, it may increase the risk of injury to the spine from mechanical stresses.

In the current study, the objective was not to assess the posture or muscular activity of adolescent dancers, but the flexibility of their spine relative to the different levels of intensity low back pain. The results showed that, regardless of the pain symptom, the dancers revealed no reduction in spinal flexibility and no difference relative to the control adolescents. Given that the dancers were teenagers who had acute symptoms of low back pain, the adaptations of spine flexibility can be more effective, but could differ with the time of practice or intensity of training. This understanding can be guided by the literature, which shows that repetitive joint movements of the spine during dance, in particular, are associated with strength overload and possible postural adjustments, especially the lumbar region supported by abdominal musculature, which can compromise the flexibility between the muscular chains: the anterior and posterior spine [6, 9, 13].

Studies with kinematics evaluations of dancers with and without low back pain, that is, assessments of the articular movement of the lumbosacral spine and the hip joint, show that pain does not influence the joint mobility of the spine of dancers with low back pain [22, 23]. Studies on ankle kinematics [20] observed that a significant decrease in the height of the medial longitudinal arch can induces decreases in the body stability during landing in jumping movements [46]. Thus, the foot support base is a main key point for integrating greater body stability and balance for dancers [7, 18].

This scientific evidence reveals that the initial understanding of the foot support base comes from the characteristic of the foot posture, which most studies identify as a pronation foot posture [7, 18, 21]. However, no scientific studies have examined this issue with regard to dancers with symptoms of low back pain, which makes it difficult to discuss the present findings. The evidence of changes in foot posture, leads to understanding of other factors, such as the intrinsic and extrinsic muscle strength of the foot [46], which in turn may be related to the time of practice and frequency of training, given that mild low back pain promoted a supine foot posture, while moderate and high levels promoted a neutral foot posture. According to Carter et al. [20], dancers are more likely to pronate their foot than rotate their knee to compensate for limited external hip rotation. Dancers with limited foot pronation ability may force additional rotation through the knee [20]. Evidently, the compensation mechanisms used by dancers to achieve the turnout depend on the dancer's functional and anatomical capacity within the closed kinematic chain, especially a dancer with low back pain. In the present study, dancers in the mild pain intensity group were associated with supine feet when compared to the moderate and severe pain intensity groups, evidencing the need for foot posture training to reduce possible knee rotation to compensate for limited external rotation of the hip.

Another important finding of this study was that the SBST score (risk of chronicity) for low back pain in adolescents who practice classical ballet was associated with the different levels of low back pain, training frequency, and time of dance practice. These findings agree with some studies that showed an association of dance practice time and training intensity [1, 23] as potential risk factors for the development of low back pain.

High intensity low back pain is a main clinical complaint among dancers who receive health services aimed at medical treatment and rehabilitation to relieve pain symptoms and avoid withdrawal from dance practice [1]. Thus, the SBST is a fundamental tool associated with the potential risk factors of chronicity for low back pain, and it may assist health professionals in planning preventive strategies to be apply during the onset of symptoms. The SBST may even change the intervention process according to the category of risk of chronicity indicated by the level of low back pain.

The important limitations of this study were: having considered the flexibility of the lumbosacral spine only in the sagittal plane and evaluating the foot posture index in only one moment (cross-sectional design). Future studies involving three-dimensional parameters for spine flexibility and foot posture adaptations with long-term monitoring, as well as the possible risk of chronicity (SBST) for low back pain through modifiable biopsychosocial factors may help in understanding the occurrence of low back pain in adolescents who practice classical ballet. In addition, the risk of chronicity for low back pain should be assessed modifiable biopsychosocial factors were in dancers with acute or chronic low back pain without physical therapy treatment.

Clinical implications: The findings of this study help the health professionals involved in preventive and conservative treatment of low back pain in adolescent’s dancers of classical ballet to understand the clinical-functional aspects and biopsychosocial risk factors for its evolution; consequently, aiding the prevention of future functional losses and musculoskeletal injuries, which can lead to withdrawals and interruptions of dance practice**.** Neglected the level of intensity low back pain can promote, over time, the appearance of musculoskeletal injuries, such as spondylolisthesis and herniated disc. Another important point was that the level of intensity moderate low back pain showed a more supine FPI support, which may favor changes in the force vectors on the lumbosacral spine, and with that, the development of increased spine pain and, consequently, chronicity. In addition, the fitness coaches responsible for training should adjust the frequency of practice according to the intensity of pain reported by the adolescents of classical ballet, taking into account their practice time.

In this study, psychosocial characteristics had an effect on the risk of chronic low back pain in adolescents from lower human development regions (Paraisópolis, São Paulo/SP). Future studies may test these characteristics against ballet practices performed in higher human development countries, given that LBP is a complex, multifactorial disease.

## Conclusion

Adolescents of classical ballet with high-intensity low back pain showed a potential risk of chronicity by the SBST. The level of intensity low back pain did not influence the clinical-functional aspects of spine flexibility in the sagittal plane, but the level of intensity moderate pain promoted changes to a more supinated foot posture. The potential risk of chronicity by the SBST also was associated with higher exposure time–frequency and time of dancing in adolescents of classical ballet.

## Data Availability

The datasets used and/or analyzed during the current study are available from the corresponding author on reasonable request.

## Abbreviations

SBST: STarT back screening tool questionnaire
FPI: Foot posture index
ANOVA: Analysis variance
